# Sowing the seeds or failing to blossom? A feasibility study of a simple ecotherapy-based intervention in women affected by breast cancer

**DOI:** 10.3332/ecancer.2015.602

**Published:** 2015-12-01

**Authors:** Ceri Phelps, Carole Butler, Alecia Cousins, Carol Hughes

**Affiliations:** 1 School of Psychology, University of Wales Trinity Saint David, Townhill Campus, Swansea SA2 0UT, UK; 2 University of Wales Trinity Saint David, Townhill Campus, Swansea SA2 0UT, UK

**Keywords:** coping, ecotherapy, intervention, distraction, nature, breast cancer

## Abstract

Engaging in nature-based activities is recognised as providing the basis for easily accessible, cost-effective interventions which can have other important physical and psychological health outcomes. The aim of the reported feasibility study was to explore the acceptability and potential psychological benefits of a simple ecotherapy-based intervention for individuals affected by cancer. A total of seven women from an existing breast cancer support group agreed to take part in the study by cultivating and customising a garden bowl for three months, maintaining a diary, and participating in a focus group at the end of the project. The analysis of the focus group data revealed four main themes that suggested that the women found engaging with the intervention to be therapeutic on a number of different levels: reflecting their cancer journey, a source of positivity, making meaning through memories, and a sense of control provided by engagement with the intervention. Engagement with the diary-writing element of the intervention, however, was not as widely endorsed by the group, as participants were even reluctant to make use of an online forum to share experiences of engaging with the intervention. Overall, the study suggests that the flexibility of level of engagement with an intervention is an important factor in developing acceptable interventions, and that the value of targeted recruitment to improve engagement with novel interventions is paramount.

## Introduction

The beneficial effects of exposure to nature-based activities, sights, and sounds on both mental well-being and physical health outcomes amongst patient and non-patient populations are becoming increasingly well-documented [1–4] including studies reporting positive outcomes in cancer populations [5, 6]. Reported benefits of engaging with nature include stress reduction [7], pain management [8], attentional restoration [9, 10], and psychological benefits [3]. Clearly, however, for individuals affected by cancer the level of active engagement with outdoor-based nature activities may be limited at certain times in their cancer journey when undergoing treatment, recovering from surgery, and experiencing the many psychological and physical side-effects of the cancer trajectory. Accordingly, there is a need to explore whether alternative mechanisms for capturing the benefits of exposure to nature can be achieved through novel yet simple interventions.

Within the field of counselling and psychotherapy, ecotherapy is the term given to the practice of encouraging individuals to engage in nature-based activities as a therapeutic method in order to gain physical and psychological benefits [11], and ecotherapy-based interventions are starting to be considered as potentially important therapeutic tools. Engaging in ‘green’ activities is now recognised as providing an easily accessible, cost-effective intervention which can improve psychological functioning, and increase individuals’ perceived ability to cope with the distress by the way of improvement in mood, confidence, and self-esteem [3, 12].

In patient populations, a growing number of studies have reported that such engagement with nature-based activities can have a positive impact upon patients’ well-being and clinical outcomes [5, 6, 10, 12]. Whilst many ecotherapy-based interventions naturally involve active engagement with outdoor spaces, there is a growing body of evidence to suggest that any nature-based sights and sounds can have positive effects on well-being and that even relatively small scale activities involving nature can produce positive effects [13–14]. Mayer *et al* [15] reported that exposure to nature, either real or virtual (an outdoor walk versus a video of natural settings) resulted in increased positive mood, a better perceived ability to reflect on problems, and greater attentional capacity. Importantly, this effect has also been found in patient populations with studies also reporting better pain control following exposure to nature-based scenes or recordings [16, 17] within the oncology field, reduced attentional fatigue after breast cancer surgery [10], and improved recovery [6]. Specifically, Yamane *et al* [18] reported that activities with plants promoted relaxation and that working with flowering plants appears to have a stronger positive effect on human emotions than non-flowering plants, with Dijkstra* et al* [19] reporting that exposure to a hospital room with indoor plants resulted in reduced feelings of stress. Park and Mattson [20] also reported that patients recovering from thyroidectomy in hospital rooms with plants and flowers had significantly shorter hospitalisations, fewer intakes of analgesics, lower ratings of pain, anxiety, and fatigue, and more positive feelings, and higher satisfaction about their rooms when compared with patients in a standard hospital room.

Despite the promises of ecotherapy-based interventions, the need for well-designed intervention research in this area has been acknowledged [21, 4]. Accordingly, the aim of this study was to explore whether the provision of a simple indoor nature-based intervention for individuals affected by cancer to make use of their home environment could potentially provide similar therapeutic benefits to those documented. Recognising the individual focus of the intervention, an online discussion forum was created as part of the study intervention to facilitate the sharing of experiences as individuals engaged with the intervention and as a tool for the research team to encourage ongoing engagement with the intervention. Accordingly, this paper reports qualitative data on the initial feasibility study exploring the acceptability and potential psychological benefits of encouraging individuals with cancer to cultivate and care for their own indoor garden bowl. The specific objectives of this study at the outset were to: 1) monitor the level of engagement with the intervention, its potential benefits and any problems or challenges raised over a period of three months, 2) explore the degree to which a website with a facility for online discussions may support the delivery and effectiveness of the intervention, and 3) identity potential subgroups who may find the intervention more or less appropriate.

## Methods

### Study design

The garden bowl intervention was designed for an individual; it is an easy to manage intervention in which participants could engage in in their own way and in their own homes. Potential participants were invited to plant, personalise, and nurture an indoor garden bowl in their own home for a period of three months and to track their experiences of engaging with the bowl through a written diary. Participants were asked to record their daily experiences of nurturing the bowl in their diaries and to capture significant moments of engaging with the bowl through photographs. Participants were also encouraged to upload photographs and to share their experiences of engaging with the garden bowl on an online discussion forum that was created for the project. Given the individual focus of the intervention, the purpose of this online forum was to enable participants to share their experience of being part of the project with other participants through the sharing of photographs or written comments.

At the end of the three months, participants were invited to a focus group in order to facilitate discussion and sharing of experiences. Focus group methodology is well-recognised methodology for exploring the psychosocial aspects of living with or at risk of cancer [22], and has been used effectively to assess the potential feasibility and acceptability of psychosocial interventions amongst specific patient groups [23]. Recognising the potential needs of the target participant group, however, participants could also choose to take part in a telephone interview rather than attend a focus group.

### Participants and recruitment strategy

Following ethical approval from the University’s School of Psychology Research Ethics Committee and the South West Wales National Health Services (NHS) Research Ethics Committee, initial recruitment efforts targeted three potential groups of participants: 1) individuals who had recently or previously been diagnosed with cancer, aged 18–40, 2) individuals who had recently or previously been diagnosed with cancer and were aged over 40, and 3) family members/carers who were supporting individuals with cancer at home. Both men and women were eligible to take part in the study. Early recruitment efforts which involved mail shots to individuals through the cancer charity funding the project, advertising in local cancer charity shops, and posters displayed in hospitals were found to be unsuccessful.

Following a talk and a demonstration from the project team to a breast cancer support group based in South Wales, seven women agreed to take part in the study. The support group offers support for men and women who have or have had breast cancer, at any stage of their breast cancer experience, and offers a number of support, information, and awareness raising activities across the South Wales region. At the support group meeting in question, only female members of the group were present. Before being asked to decide whether to consent into the study, the support group members attended a brief demonstration from the research team (including a qualified horticulturalist) and were shown how to look after their indoor garden bowl and given ideas of how they could personalise or customise their bowl if they wished to. Following this demonstration, seven members of the support group agreed to take part in the study. All of these women had completed cancer treatment for breast cancer; some women were many years past diagnosis, others more recently completing treatment. These participants were provided with the bowl complete with compost and three starter plants and a ‘helpful hints’ factsheet providing information about how to nurture their bowl on a daily basis. Participants were then able to take their bowl home with them following this demonstration. It was clear at this stage of the project that these participants were reluctant to engage with the online forum element of the project and therefore this was abandoned.

### Engagement with the intervention and data collection

The seven female participants were asked to tend to their indoor bowls for a three month period and were also given the option to keep a personal journal of their experiences. During this period, members of the research team maintained contact with the participants who shared their experiences and pictures via email. At the end of this period, the participants attended a focus group discussion which explored issues such as participants’ previous gardening experiences, their thoughts and feelings regarding the project, and their interactions with the garden bowl. In addition to this, other issues addressed included what aspects of the project they enjoyed or did not enjoy and their thoughts and feelings regarding similar future research. A telephone interview was conducted for one participant who was unable to attend the final group discussion.

### Analysis

The group discussions was tape-recorded, transcribed, and thematically analysed by the project research assistant in line with Braun and Clarke [24]. Emerging issues were identified by studying the transcripts repeatedly by hand and exploring possible meanings before entering them into NVivo 9 software. Segments of text were coded and grouped under different themes. Axial coding was used to link subordinate themes to the main themes and other subordinate themes. The transcripts were read independently by two researchers and the main thematic analysis validated through discussion. Participant validation was achieved by feeding back the key themes to participants from the first meeting through a summary of results. All participants were asked to confirm that they were an accurate representation of their discussions.

## Results

The analysis of the focus group data revealed the following four main themes that suggested that the women found engaging with the intervention to be therapeutic on a number of different levels: reflecting their cancer journey, a source of positivity, making meaning through memories, and a sense of control provided by engagement with the intervention.

**Reflecting their cancer journey:** The bowl appeared to evoke reflections about their own cancer journeys with one lady talking about how the bowl represented to her the fragility of life.

‘I just think the whole of life was in that bowl … I went away for a weekend and when I came home lots of the plants had died and I just thought you know it’s just the fragility of life really and so that brought it back to our scenario’ (P3).

She also went on to read out an excerpt from her diary that she had also been keeping during the study period that further demonstrated the extent to which the way in which the day-to-day changes in the flowers within the bowl were seen as reflecting her own daily challenges.

‘I came home from work today to find that the tete a tete flowers have grown so tall that they are leaning in all directions. This summed up the way I was feeling today - a bit tired and frayed round the edges. I turned the bowl around and let it lean in a different direction’ (P3).

For some participants the bowl appeared to take on an almost ‘human’ dimension, with comments such as the bowl *‘enjoying the sunshine’* and *‘the strawberries are going ‘ah no I can’t be bothered’’ (P4).*

Importantly, participants agreed that the nature of the intervention is appropriate for anyone at any stage of their cancer journey,

‘And you can watch it grow and you have to look after it …. And I think it doesn’t matter whether you’ve just been diagnosed or been a year or five years or ten years down the road it could be beneficial. It could help you deal with whatever you have left behind’ (P4).

There appeared to be a clear perception within the group that they often felt that those a few years past treatment were not considered eligible to access other psychosocial support offered by charities or wider cancer services *‘we maybe think well perhaps it is only just those immediately diagnosed’ (P7).*

‘We’re all even around this table at all very different stages away from our initial diagnosis um and I think everyone has gained in different ways and it’s this I think it’s the acceptance that yes we can all gain [from engaging with this intervention] at different stages’ (P7).

**A source of positivity:** Some participants talked about the positive feelings of hope, pride, and responsibility that looking after the bowl created. One lady talked about how agreeing to take part in the project and having to look after the bowl gave her impetus and energy for engaging in other gardening activities.

‘The bowl kick started this year’s gardening again and I thought ‘come on I can get out here and do that’, and as soon as you get out there and do something you remember how good it makes you feel anyway, so um so yea - re-energising that bowl was’ (P7).

As the discussions evolved it became apparent that creating an attractive garden bowl and nurturing these bowls on a daily basis resulted in a sense of pride and achievement. *‘And when I go outside I go “doing well, doing well”’ (P4). *A number of participants talked about how they moved the bowls inside and outside of the house depending on the optimum conditions, with a clear sense of responsibility and care:

‘Mine was indoor now it it’s outside and I thought ‘oh it can’t just sit on the ground’ it is too important for that, so I had a ceramic pot I turned it upside down so it’s got its own little stand’ (P3).

For another participant who was not a regular gardener, the positive feedback she received from others about the bowl gave her a sense of pride and achievement;

‘It was the first bowl I’d ever planted and I thought Wow I’ve done that, that looks smashing! And somebody came and went ‘that’s great where did you get that from’? Which I have to admit made me feel really good’ (P6).

Encouragingly, another participant talked about how engaging with the garden bowl had encouraged them to plan more nature based activities in the future *‘I am hoping as a result of having my bowl, to do some more bedding plants and to do some more with it this year’ (P5).*

**Making meaning through memories:** A number of participants personalised their bowls with mementos or items of personal significance. One participant talked about decorating her bowl with a chocolate Easter rabbit, another placed a palm cross on the bowl on Palm Sunday, whilst another planted purple for the jubilee. These efforts to personalise the bowls to mean different things on different occasions appeared to keep participants interested and engaged with the intervention.

‘Mine was sort of a mobile bowl ….when the frost was around I brought it in at night and the sun was out in the morning put it out on my garden table so it really travelled miles …at the moment it’s got everything purple in it it’s almost like a Royal Jubilee… it’s been a joy it really has..’ (P2).

Another lady talked about how she had decorated her bowl with mementos from a day at the beach, and this again appeared to create further engagement with the intervention linked to positive memories.

‘I remember going to the beach…with the dog and picking up some shells and put them in my bowl and every time I see them in the bowl now I remember that day we had a lovely walk on the beach whereas we’d have forgotten all about it….’ (P5).

### A sense of control from engaging with the intervention

It was clear from the discussions that participants enjoyed the flexibility of engaging with the intervention but also the element of control which they had over it when they engaged with either the diary or gardening elements of the intervention.

‘And you can work at your own pace at it like you said you know couldn’t plant em til the next day so that fitted in with your time scale um you know when you came home from work and you wait til the weekend to do the diary it fits in with uh with the rest of your life so it’s something you can do’ (P6).

‘I think the gardening is much more approachable…that bowl and the different options of the bowl…its something you can look after as much or as little as you want and then look at your result’ (P4).

The ease with which participants could care for the bowl appeared to be a particularly positive element:

‘You can do as much or as little with it so if you are not a keen gardener…. I can deal with the herbs …and they don’t need a lot of attention you just say ‘Hi you’re doing well, oh let me turn you around’….. It’s this question of life I think and maybe a little bit of responsibility’ (P4).

**Unease with diary-writing element:** Only three participants had attempted to keep a diary alongside caring for the garden bowl, and whilst these participants reported positive experiences with writing in their diary, it was clear that this was also a potentially off-putting part of the project for many participants:

Facilitator 2: ‘I was wondering then what about how you felt about the idea of writing in a diary at the time when we asked you’?

‘I did well in the beginning I’ve just I must admit I haven’t done anything since March But I started off well… I put a few thoughts and feelings down about my situation and whatever so it was useful but I think that life took over then. It wasn’t that I didn’t have time to do it, it was just that other things cropped up’ (P3).

‘I have to say that frightened me off the idea of taking part completely because I’m not a person to do that sort of thing - I thought I’ve got to do a diary I can’t do it’ (P6).

In contrast to the positive attitudes expressed towards the flexibility of level of interaction with the garden bowl, the expectation of regular engagement with the diary was also potentially off-putting part, with one participant explaining that: *‘It wasn’t something that I could come home from work and think oh I’ve gotta write something down but on weekends I would have given it you know my time’ (P5)*.

## Discussion

Whilst modest, the results of this study suggest that those who engaged with the intervention reported similar effects as reported in other studies involving nature-based activities. These reported benefits included feelings of relaxation, improvements in mood, increased confidence and feelings of control, and improved self-esteem [3, 18, 25]. Importantly, the findings of the current study suggest that these positive effects appear to be attainable through unique interventions involving alternative means of engaging with plants and nature for those who may not be in a position to actively engage with the physical outdoor environment. In the current study, engaging with the intervention clearly evoked feelings of joy, positivity, reflection, and control. The role of such positive emotions in helping individuals cope with the cancer experience has been well-documented in previous literature [26, 27], and the results of this study suggest that simple ecotherapy-based interventions may offer a novel mechanism to promote such coping responses.

The results of this feasibility study offer some useful insight into the processes of engaging potential participants in novel intervention studies that may not appear to have a direct impact upon their clinical trajectory. Having a perception of an element of control is an important mechanism when coping with illness and engaging with the therapeutic intervention process [28, 29]. In this study, the flexibility of the level of required engagement with the intervention appeared to be an important element of the acceptability of the current intervention through providing participants with a sense of control over this small aspect of their lives. Our findings suggest therefore that the flexibility of level of engagement with an intervention is an important factor in developing acceptable novel interventions and may be particularly important for individuals at different stages of their cancer journey. In the current study all the participants had completed cancer treatment; some women were many years past diagnosis, others more recently completing treatment. There was a clear suggestion amongst participants that individuals who were several years post diagnosis and treatment are often forgotten about in term of the provision of psychosocial support. An additional consideration is that no men took part in the study and therefore we do not know whether the therapeutic benefit or level of engagement with such an intervention would have been the same.

Whilst the main focus of the study was to explore the possible therapeutic effects of nurturing an indoor garden, it is recognised that there may also have been potential benefits experienced as a result of keeping a journal, or from sharing personal experiences via the online forum. Studies have found that expressive writing or journal therapy can significantly improve the health and psychological well-being of people with cancer [30, 31]. Such interventions are not considered universally acceptable or beneficial however, and in the current study engagement with the diaries did not appear to underpin the active component of the intervention. Similarly, none of the participants wished to engage with the online forum. The latter observation may reflect the older age range of the participants who took part in the study, with literature suggesting younger people may be more willing to make use of online interventions for psychosocial support [32, 33]. It appears plausible that the online discussion forum element of the original proposal may well have been a barrier to participation for many potential participants. Whilst cancer online support groups have been found to help people with cancer cope more effectively [34, 35], it is clear that many people still can be reluctant or suspicious of engaging with such online forms of support [36]. It is possible therefore that attempting to incorporate more than one type of intervention or activity in an overall study design may not be the best way of promoting engagement, and can clearly produce difficulty and confounding variables if subsequently planning to evaluate the active components of an intervention programme. Important to note, however, is that clearly in the context of this study a pre-existing support group may well have faciliated ongoing engagement with the intervention beyond the opportunity to take part in a focus group.

Whilst those who took part reported finding engaging with the intervention to be therapeutic on a number of different levels, it is also clear that research teams need to do more to promote such intervention studies to potential participants in order to gain wider commitment. Difficulties in recruiting for this project shows the importance of the ‘personal touch’ in gaining enthusiasm and commitment for such ecotherapy-based projects in a population often bombarded with requests for research participation. The participants who chose to take part in this study were all highly engaged and enthused throughout the process, and it was clear that meeting the research team and having received the demonstration in advance of consenting to the study had made a significant impact on their engagement with this study.

## Conclusion

Overall, the results of this study have provided valuable insight into not only the potential benefits of ecotherapy-based interventions amongst populations affected by cancer, but also some of the potential challenges of engaging such populations in potentially beneficial activities within the context of a research project. Ultimately, the results of this study will inform larger studies of the effectiveness of such ‘eco-interventions’ with clear potential for future support and care initiatives for individuals affected by cancer and their families, and also providing information on how such individuals can be successfully engaged and supported through this process.

## Conflict of interests

This study was funded through the Tenovus Innovation Grant Scheme 2010.
